# Predicting Pharmacokinetics of Active Constituents in *Spatholobi caulis* by Using Physiologically Based Pharmacokinetic Models

**DOI:** 10.3390/ph17121621

**Published:** 2024-12-03

**Authors:** Xiaoyan Liu, Ruihu Du, Tao Zhang, Yingzi Li, Ludi Li, Zheng Yang, Youbo Zhang, Qi Wang

**Affiliations:** 1 Department of Toxicology, School of Public Health, Peking University, Beijing 100191, China; xyl@bjmu.edu.cn (X.L.); duruihu@bjmu.edu.cn (R.D.); liyingzi@bjmu.edu.cn (Y.L.); liludi@bjmu.edu.cn (L.L.); 1610306217@bjmu.edu.cn (Z.Y.); 2 State Key Laboratory of Natural and Biomimetic Drugs, Department of Natural Medicines, School of Pharmaceutical Sciences, Peking University, Beijing 100191, China; ybzhang@bjmu.edu.cn; 3 Key Laboratory of State Administration of Traditional Chinese Medicine for Compatibility Toxicology, Beijing 100191, China; 4 Key Laboratory of Toxicological Research and Risk Assessment for Food Safety, Beijing 100191, China

**Keywords:** physiologically based pharmacokinetic model, pharmacokinetic characteristics, oral administration, extrapolation

## Abstract

Background/Objectives: Spatholobi Caulis (SPC) is a medicinal plant that mainly grows in China and Southeast Asian countries and commonly used in clinics; the pharmacokinetic characteristics in humans need to be determined. This study was to establish the physiologically based pharmacokinetic (PBPK) models of multiple active constituents from SPC in rats, and predict the pharmacokinetic properties of rats with different dosages and extrapolated to humans. Methods: The parameters were collected based on our previous study and by prediction using ADMET Predictor software predict. The PBPK models for 3′-methoxydadizein (**1**), 8-*O*-methylretusin (**2**), daidzin (**3**), and isolariciresinol (**4**) administered orally to rats were established using GastroPlus software. These models were employed to simulate the pharmacokinetic properties in rats across various dosages, and subsequently extrapolated to humans. The calculated parameters including *C_max_*, *T_max_*, and *AUC* were compared with observed values. The accuracy of the PBPK models was assessed using fold-error (FE) values. Result: The FE values ranged from 1.03 to 1.52, meeting the PBPK model regulations where FE should be less than 2. The sensitivity analysis focusing on the absorption amount and *AUC_0__→t_* of these four constituents in humans was also conducted. These results confirm the successful establishment of PBPK models of these four constituents from SPC in this study, and these models were applicable to predict pharmacokinetics across various doses and extrapolate across species. Conclusions: The PBPK models of four constituents can be used to predict the pharmacokinetic characteristics in humans after oral administration of SPC and provide useful data for safe and rational medication in clinical practice.

## 1. Introduction

Spatholobi Caulis (SPC), derived from the dried tuber of *Spatholobus suberectus* Dunn [1], is one of the medicinal plants that grows in China, Japan, and other countries in Southeast Asia, especially has been commonly used in traditional Chinese medicine (TCM). It is commonly used, in combination with other TCMs, to treat dysmenorrhea, amenorrhea, and rheumatoid arthritis [2,3,4]. Modern pharmacological studies have revealed that SPC exhibits diverse effects, including antioxidant and anti-inflammatory properties, regulation of lipid metabolism, and anti-tumor activities [5,6,7,8]. Various constituents enriched in SPC, particularly flavonoids, play pivotal roles in its pharmacological effects [8,9,10]. In clinical practice, oral administration is one of the primary routes of drug delivery for the TCM, the constituents of which can be absorbed into the blood through the gastrointestinal tract, and distributed to target organs via blood circulation to exert their effects; after being metabolized in the liver, these constituents or their metabolites are excreted eventually by the kidneys or other organs. The in vivo pharmacokinetics of TCM have thus become crucial for determining its efficacy and safety. Currently, studies have reported the pharmacokinetics of individual constituents such as ononin [11], formononetin [11], genistein [12,13], and daidzein [14], which are monomers from SPC. However, only two studies have investigated the pharmacokinetics of multiple components from SPC in rats [15,16], and there is a lack of pharmacokinetic data in humans. The characteristics of “multi-component, multi-target, and multi-pathway” in TCM pose significant challenges in understanding their mechanisms of action. Pharmacokinetic evaluations of multiple active constituents from SPC, and the extrapolation of pharmacokinetics from animals to humans, are critically important for rational clinical medication.

The physiologically based pharmacokinetic (PBPK) model utilizes knowledge of human physiology, biochemistry, and drug characteristics to mechanistically and quantitatively describe drug absorption, distribution, metabolism, and excretion through fitting and simulation methods. This model can simulate the dynamic changes in pharmacokinetics within organisms and facilitate extrapolation across various drug doses, dosage forms, and even different species [17,18,19]. Due to its unique capabilities in prediction and extrapolation, the PBPK model has been extensively utilized in pharmacokinetic studies, both in vitro and in vivo and in data mining, drug design, drug-drug interactions, and toxicity and safety evaluations [20,21]. Recognized by the Food and Drug Administration (FDA) as a convenient research tool for pharmacokinetic studies [22], the PBPK model has also been applied in TCM [23]. By simulating the body’s physiological environment, this model not only explains the absorption of TCM constituents in the human body but also predicts their pharmacokinetic behavior.

The pharmacokinetic behaviors of SPC at various dosages in humans remain unclear and warrant further investigation. Based on our previous study on SPC pharmacokinetics, four representative constituents with significant activity [16], 3′-methoxydadizein (**1**), 8-*O*-methylretusin (**2**), daidzin (**3**), and isolariciresinol (**4**) were selected to predict the pharmacokinetic behaviors at different doses for the first time. ADMET Predictor software was used to estimate relevant parameters and GastroPlus software to construct PBPK models for these four constituents. The pharmacokinetic results for different doses and multiple dosing were predicted and subsequently verified through experiments in rats. Subsequently, the rat pharmacokinetic data were extrapolated to humans to simulate human pharmacokinetic characteristics. This study supports the research and development of innovative drugs and provides scientific data for the safe and rational clinical use of medications.

## 2. Results

### 2.1. Establishing the PBPK Models of Active Constituents

#### 2.1.1. Parameterization

**Figure 1:**
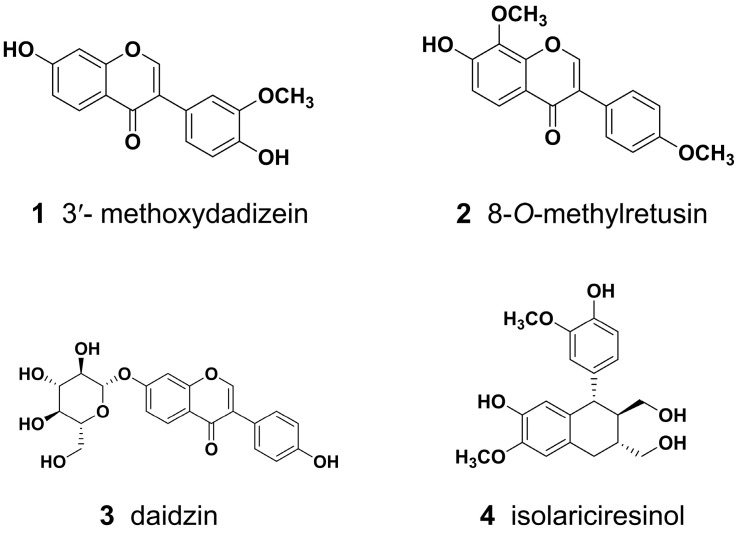
The four active constituents extracted from SPC.

The main parameters, molecular weight (*MW*), the logarithm of octanol: water partition coefficient (*Log P*), the negative logarithm of the acid ionization constant (*pKa*), apparent partition coefficient (*Log D*), solubility, apparent permeability coefficients (*P_app_*), blood-plasma concentration ratio (*R_bp_*), plasma free fraction (*f_up_*), clearance (*CL*), and steady-state volume of distribution (*Vss*) of rats and humans used to construct the PBPK model of four constituents from SPC (the structures are shown in Figure 1) in rats are shown in Table 1. The *P_app_* values were determined by our previous study using the Caco-2 cell monolayer model to simulate the intestinal permeability of these constituents [24,25]. Solubility and respective pH were predicted by GastroPlus software, and other parameters were predicted by ADMET Predictor software. The *K_p_* values of four constituents in different tissues are listed in Table 2. These parameters not only affect drug absorption, distribution, and metabolism, but also play a decisive role in the quality of the model, making them key parameters that cannot be ignored in PBPK modeling.

#### 2.1.2. Construction and Validation of PBPK Models

The parameters of the four constituents presented in Table 1 were imported into GastroPlus software in the module of the drug in rats by oral administration. The doses of constituents **1**–**4** were set to 0.062, 0.11, 0.71, and 1.31 mg/kg by referring to our previous pharmacokinetic study [16], respectively. The PBPK models of these four constituents were constructed after the parameters were optimized repeatedly.

**Figure 2:**
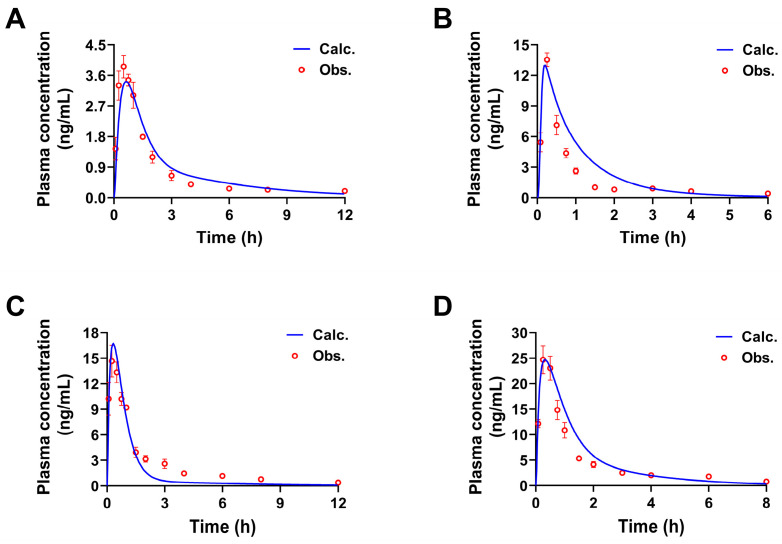
The concentration-time curves of four constituents from SPC in rats (p.o.). observed and simulated by PBPK models. The observed and calculated concentration-time curves of 3′-methoxydadizein (**A**), 8-*O*-methylretusin (**B**), daidzin (**C**), and isolariciresinol (**D**), respectively (Mean ± SD, *n* = 5).

**Figure 3:**
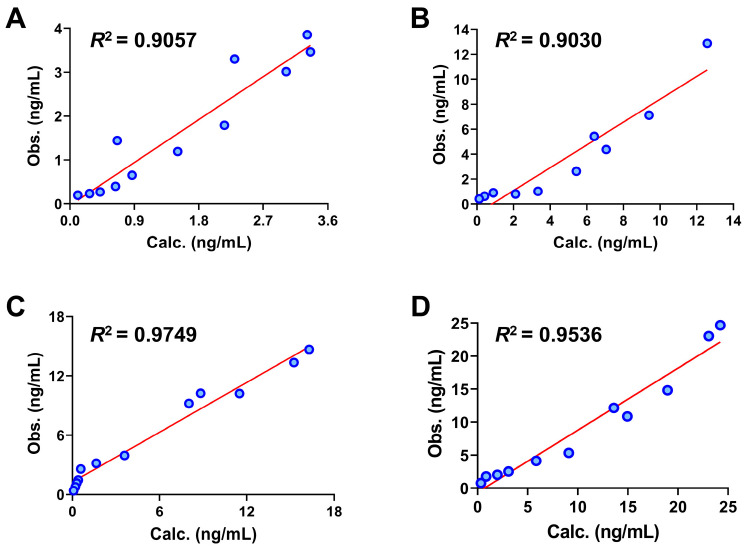
Correlation between observed and calculated values of four constituents in rats by gavage SPC. Correlation between observed and calculated values of 3′-methoxydadizein (**A**), 8-*O*-methylretusin (**B**), daidzin (**C**), and isolariciresinol (**D**), respectively. Note: The hollow circles represent the intersection of the two values; the solid line represents a linear regression.

The calculated concentration-time curves were in good coincidence with the observed curves to a certain extent (Figure 2), and both fit the linear mathematical regression model by analyzing their correlation (Figure 3). *R*^2^ was in the range of 0.9030 to 0.9749 (the limit of the value was 0.8 [26,27]), indicating a reasonable fitting effect. By comparing the calculated and observed pharmacokinetic parameters *C_max_*, *T_max_*, *AUC_0__→t_*, and *AUC_0__→__∞_* of each constituent, it could be seen (Table 3) that the two values were similar and had no difference in the order of magnitude. The fold error (FE) is one of the indicators to evaluate the fit degree of observed and calculated values of PBPK models, the FE values of the listed parameters ranged from 1.03 to 1.52, less than 2 [28], indicating that the PBPK models have a good fitting effect. Hence, the PBPK models were well established by assessing the *R*^2^ and FE values and could be used to accurately reflect the pharmacokinetic processes of these constituents from SPC after oral administration to rats.

#### 2.1.3. Sensitivity Analysis of Parameters

**Figure 4:**
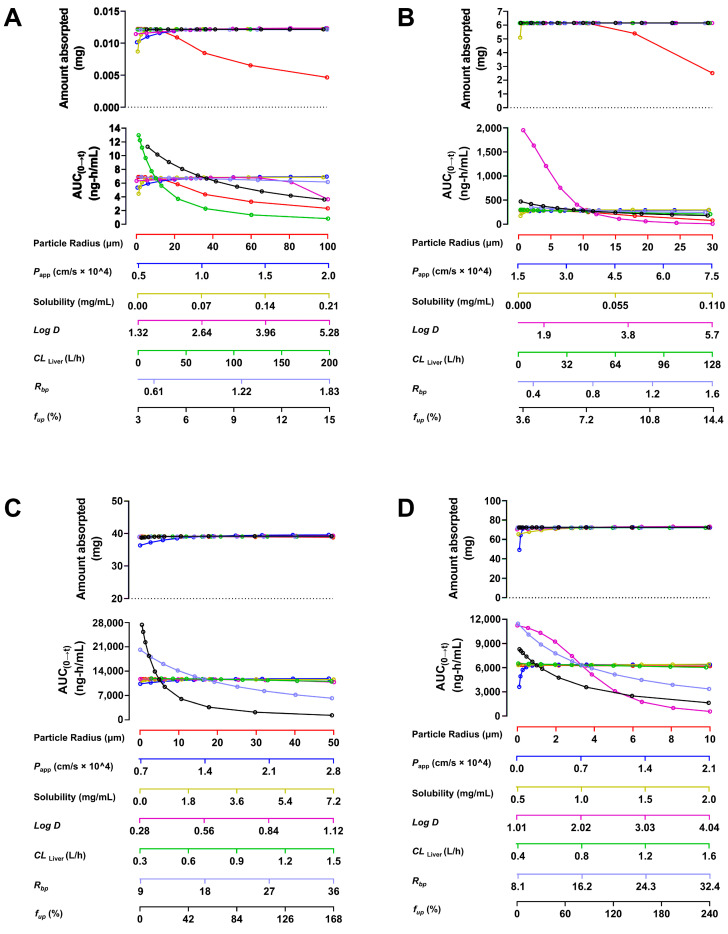
Sensitivity analysis curves of four constituents in humans. The sensitivity of the absorption amount and *AUC_0__→t_* of 3′-methoxydadizein (**A**), 8-*O*-methylretusin (**B**), daidzin (**C**), and isolariciresinol (**D**) to changes in particle size of constituents, *P_app_*, Solubility, *Log D*, *CL_Liver_*, *R_bp_*, and *f_up_*.

We conducted sensitivity analysis focusing on the absorption amount and *AUC_0__→t_* of these four constituents in humans, examining the parameters such as particle size of constituents, *P_app_*, Solubility, *Log D*, *CL_Liver_*, *R_bp_*, and *f_up_* that influence drug absorption and distribution. The results are presented in Figure 4.

As shown in Figure 4, the absorption of 3′-methoxydadizein (A) and 8-*O*-methylretusin (B) in blood exhibited significant sensitivity to particle size; the larger particle size of the constituents makes it difficult to be absorbed into the blood, while these two constituents showed no notable sensitivity to other parameters. The *AUC_0_*_→*t*_ of all four constituents demonstrated high sensitivity to *f_up_*. Additionally, 3′-methoxydadizein (A) was sensitive to *CL_Liver_*, 8-*O*-methylretusin (B) and isolariciresinol (D) were sensitive to *Log D*, daidzin (C) and isolariciresinol (D) were sensitive to *R_bp_*. These results suggest that these parameters impact the absorption, distribution, and metabolism of these four constituents in the human body to varying degrees. Therefore, adjusting and optimizing these parameters can enhance the alignment between predicted and observed values.

### 2.2. Pharmacokinetic Predictions of the Four Constituents in Rats at Different Doses

**Figure 5:**
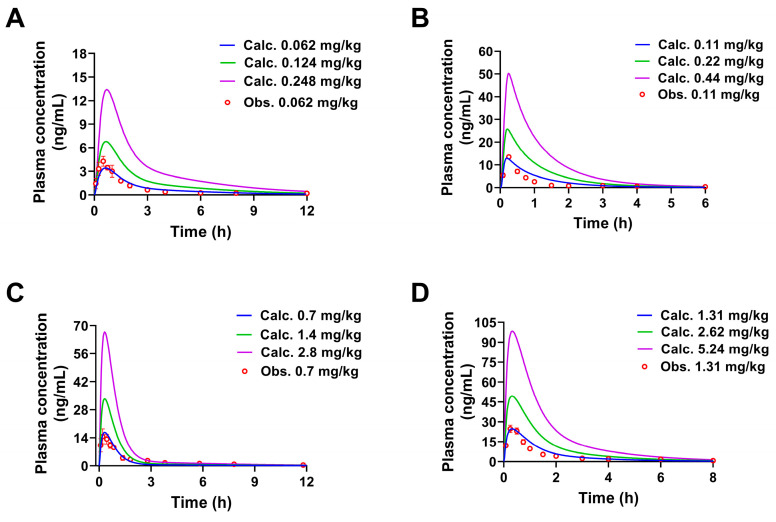
The concentration-time curves at different doses of four constituents in rats by p.o. simulated by PBPK models. The simulated concentration-time curves at different doses of 3′-methoxydadizein (**A**), 8-*O*-methylretusin (**B**), daidzin (**C**), and isolariciresinol (**D**).

**Figure 6:**
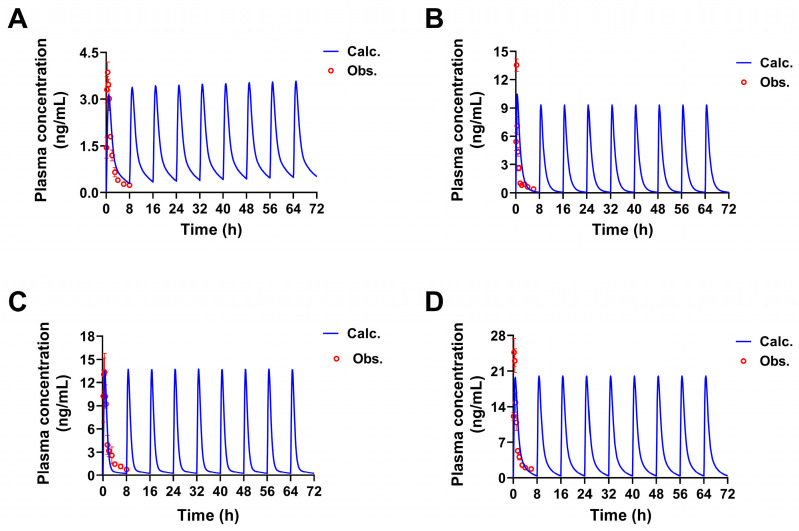
The multiple concentration-time curves of four constituents in rats by p.o. simulated by PBPK models. The simulated multiple concentration-time curves of 3′-methoxydadizein (**A**), 8-*O*-methylretusin (**B**), daidzin (**C**), and isolariciresinol (**D**).

The pharmacokinetic characteristics of the four constituents in SPC at twice (60 g crude drug/kg body weight), quadruple, and multiple the previous dose (30 g crude drug/kg body weight) given to rats by gavage were predicted by the established PBPK models. As shown in Figure 5, when the oral dose was gradually increasing, the *T_max_* of constituents did not change significantly, but *C_max_* almost increased exponentially. The results of administering multiple doses every 8 h showed the pharmacokinetic characteristics of rapid absorption and metabolism for these four constituents (Figure 6), with no accumulation of the constituents within this time interval. These predicted results showed that the four constituents could be absorbed into the blood and eliminated rapidly, even at increased doses. This result can provide reference significance for the safety range use of SPC.

To validate the accuracy of PBPK models in predicting the pharmacokinetics of SPC constituents at different oral doses, the pharmacokinetic behavior of these four constituents from SPC in rats were analyzed by UFLC-MS/MS. The parameters were calculated using Drug and Statistics Software version 2.0, and then these validated values (Val.) were used to check the accuracy of the Calc. values and evaluated by FE.

**Figure 7:**
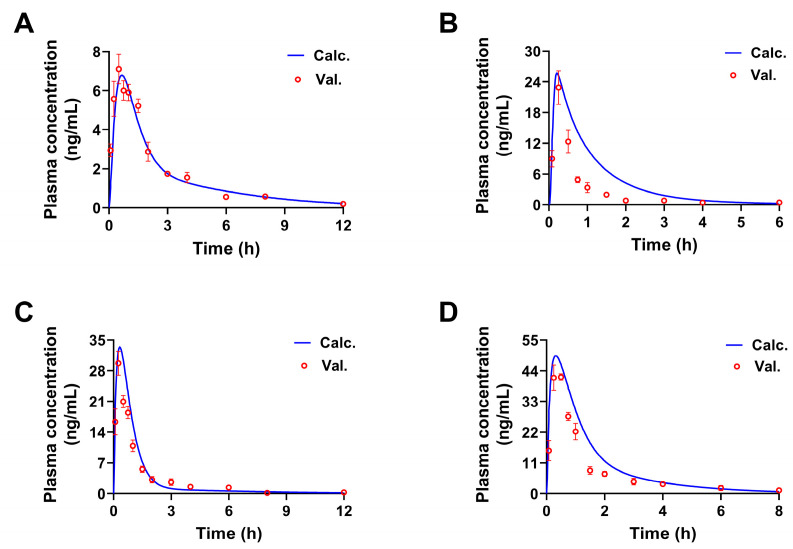
The concentration-time curves of four constituents from SPC in rats (60 g crude drug/kg body weight by p.o.). validated and simulated by PBPK models. The validated and simulated concentration-time curves at twice doses of 3′-methoxydadizein (**A**), 8-*O*-methylretusin (**B**), daidzin (**C**), and isolariciresinol (**D**) (Mean ± SD, *n* = 5).

**Figure 8:**
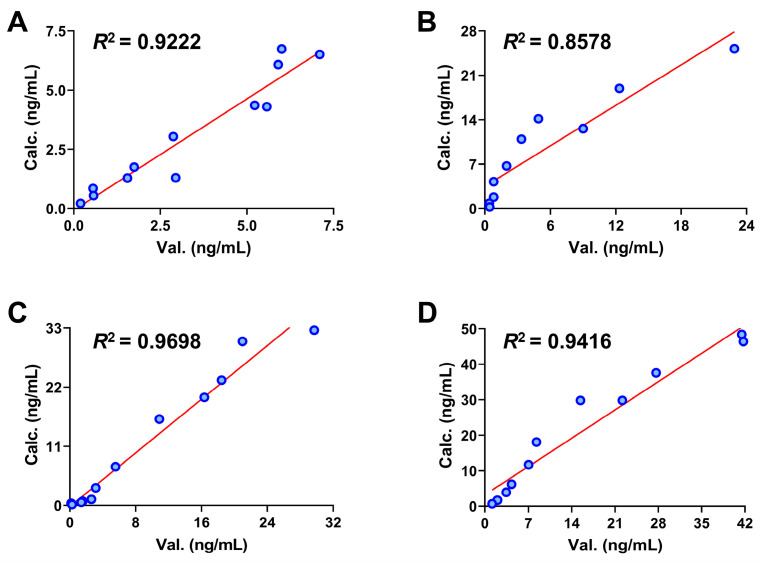
Correlation between calculated and validated values of constituents **1**–**4** in rats by gavage 60 g crude drug/kg bodyweight of SPC. Correlation between calculated and validated values of 3′-methoxydadizein (**A**), 8-*O*-methylretusin (**B**), daidzin (**C**), and isolariciresinol (**D**). Note: the hollow circles represent the intersection of the two values; the solid line represents a linear regression.

Compared with the predicted values, when the doses of 3′-methoxydadizein (**1**), 8-*O*-methylretusin (**2**), daidzin (**3**), and isolariciresinol (**4**) were 0.124, 0.22, 1.42, and 2.62 mg/kg, respectively (Twice of the primary doses), the trend of the concentration-time curves obtained from experiments were consistent with the calculated by PBPK models. *R*^2^ was in the range of 0.8578 to 0.9698 (Figure 7 and Figure 8), and the FE values obtained by Val. and Calc. parameters of *C_max_*, *T_max_*, *AUC_0__→t_*, and *AUC_0__→__∞_* were all less than 2 (Table 4). These results indicated that the established PBPK models are acceptable and can be used to predict the pharmacokinetic characteristics of the constituents in SPC at different doses.

### 2.3. Pharmacokinetic Predictions of the Four Constituents in Humans

**Figure 9:**
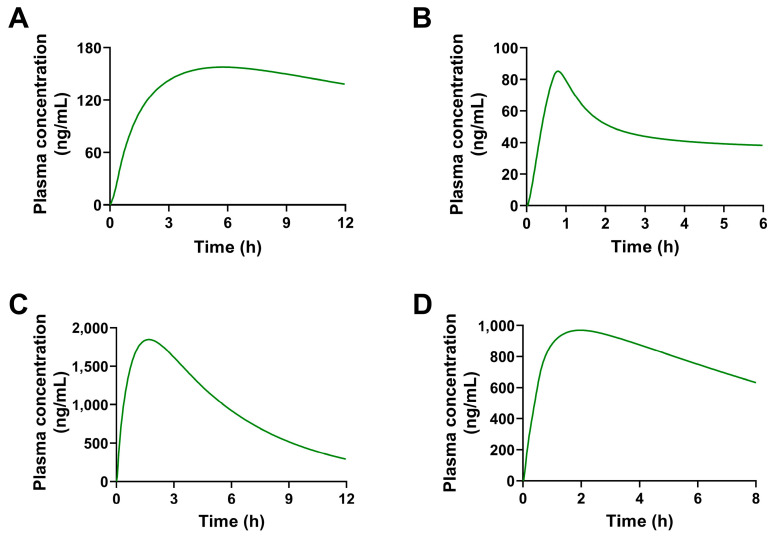
The plasma concentration-time curves of four constituents in humans by p.o. simulated by PBPK models. The simulated concentration-time curves in humans of 3′-methoxydadizein (**A**), 8-*O*-methylretusin (**B**), daidzin (**C**), and isolariciresinol (**D**).

Based on the PBPK models of these four constituents in rats, a corresponding model for oral administration at the same dosage in humans was established. The predicted concentration-time curves in humans are shown in Figure 9 and the parameters are shown in Table 5. When the concentrations of 8-*O*-methylretusin (**2**), daidzin (**3**), and isolariciresinol (**4**) reached the peak values, the concentration decreased obviously with the prolonging of time. Compound 3′-methoxydadizein (**1**) did not show such an apparent elimination trend; whether it was due to the properties of this constituent or other reasons needs to be further verified by experiments.

## 3. Discussion

SPC has a long medicinal history and extensive pharmacological effects [1,2,3,4]. While the pharmacology and pharmacodynamics of SPC have been the focus of current research, pharmacokinetic studies, particularly in humans, are rarely reported and warrant further investigation. This study investigated the pharmacokinetic characteristics of 3′-methoxydadizein (**1**), 8-*O*-methylretusin (**2**), daidzin (**3**), and isolariciresinol (**4**) from SPC and provided a convenient method for the pharmacokinetic study in different species. The PBPK model is widely used in the fields of drug screening, pharmacokinetics or toxicokinetics, drug-drug interactions, and toxicological risk assessments [29,30,31]. In the process of establishing the PBPK models of four constituents from SPC, based on the slight secondary absorption peaks observed for these four constituents at 3 h (8-*O*-methylretusin) or 6 h (3′-methoxydadizein, daidzin, and isolariciresinol), it is preliminarily inferred that enterohepatic circulation may be involved in the pharmacokinetic processes of these constituents in rats; the enterohepatic circulation module in GastroPlus software has been enabled to accurately reflect the pharmacokinetic changes of constituents in rats, and also to correct the accuracy of these PBPK models and reduce the fitting deviation. Compared with the pharmacokinetic experiment, the established PBPK model can be used to predict the pharmacokinetics of different doses or species.

As mentioned in some reports and the Chinese Pharmacopoeia Commission [1,4,32], SPC contains multiple active constituents and exhibits significant pharmacological activities in treating inflammation-related diseases. Due to the variety of constituents in herbal drugs, the pharmacokinetic characteristics of each compound are influenced by each other when the herbal drugs enter the body in the form of “constituent group” [33,34]. Consequently, the pharmacokinetic characteristics of these constituents may differ from those observed when they are administered individually [16,35]. Furthermore, the clinical dosage of SPC ranges widely, from 7.5 g to 45 g [36], making pharmacokinetic studies with different dosages more frequent and the experimental process more complex.

The four constituents we selected are representative active components of SPC and their pharmacokinetic processes have been studied in our previous study [16]. Although studying the pharmacokinetic characteristics of more constituents in SPC extract would better represent the overall pharmacokinetics of this extract, the chemical constituents of SPC extract is highly diverse, making it impractical to characterize the pharmacokinetics of each compound. Therefore, four representative constituents were selected and their PBPK models were established based on the concentration-time curve profiles with good validation (*R*^2^ and FE values were all acceptable), this approach can reflect the PBPK characteristics of SPC extract to some extent. Moreover, their fitting degree to the PBPK models was high, enhancing the model’s accuracy. The established PBPK models of SPC containing different constituents can predict the pharmacokinetic process in a manner close to clinical practice and adhere to the 3R (Reduction, Replacement, and Refinement) principle, thereby avoiding more complex experiments.

The parameters we selected for modeling, including both constituent-specific properties and physiologically based pharmacokinetic parameters, influence the processes of absorption, distribution, metabolism, and elimination of these four constituents in SPC. Sensitivity analysis has revealed that parameters such as *f_up_*. *CL_Liver_*, *Log D*, and *R_bp_* can significantly affect the absorption capacity of the constituents, introducing potential uncertainties into PBPK modeling. These parameters should be given particular attention during the modeling and optimization process. The Monte Carlo simulations or population-based modeling can be used to account for parameter variability, or use some surrogate data or analog constituents for initial estimates [37,38].

Although the PBPK model of these four constituents of SPC provides a more intuitive and convenient approach for predicting the pharmacokinetics of SPC in humans, this study has certain limitations when compared to the actual results. First, the predicted results by PBPK model are an integration of multiple simulation processes, including protein binding, tissue distribution, body composition, blood flow rates, and enzymatic reactions in metabolic tissues, which inherently differ from actual conditions. Second, the PBPK model for SPC established in this study primarily relied on published or internally validated equations to construct the model. Sensitivity analysis can evaluate the impact of individual parameters, screening out input parameters with large uncertainties, which can help confirm the priority of data collection or experimental research, thereby reducing the uncertainty of these input parameters and model outputs; however, the variable ranges of these parameters are not explicitly defined. Furthermore, as an extract of TCM, the pharmacokinetics of SPC lack sufficient clinical data, which imposes constraints on the application of the PBPK model. Even though there are certain differences between the results predicted by the PBPK models and the observed results, the difference may be more obvious in the prediction results of humans; our constructed PBPK models provide valuable reference points for the pharmacokinetic evaluation of multiple constituents administered together, laying the foundation for further predictions of the pharmacokinetic processes of active constituents from medicinal plants in multiple oral administrations or special populations. 

## 4. Materials and Methods

### 4.1. Materials

#### 4.1.1. Parameters for the PBPK Model

The ADMET Predictor ^TM^ (10.0) software was used to predict some required parameters; the GastroPlus ^TM^ (9.8.2) software (Simulations Plus, Inc., Lancaster, CA, USA) was used to establish the PBPK model and simulate different pharmacokinetic behaviors of these four constituents.

#### 4.1.2. Experimental Verification Materials

For the experimental verification of the pharmacokinetic behaviors of four constituents predicted by the PBPK models in rats after oral administrated SPC, the materials used in this experiment are listed in Supplementary Data.

### 4.2. Methods

#### 4.2.1. Model Parameters and Assumptions

The physicochemical and biochemical parameters of these constituents from SPC were collected, including molecular weight (*MW*), solubility, dissociation constant (*pKa*), oil-water partition coefficient (*LogP*), plasma free fraction (*f_up_*), blood-plasma concentration ratio (*R_bp_*), etc. Based on the results of our previous ADME experiments of SPC [16], parameters such as blood concentration (*C_max_*), clearance (*CL*), and area under the curve (AUC) of the active constituents of SPC were collected. Additionally, we incorporated the CYP enzyme system, which influences the metabolism of constituent, into the establishing of PBPK model.

Based on the predictions from the ADMET Predictor software, these constituents are primarily metabolized by CYP1A2 or CYP2C9 in vivo, and we assumed that no involvement of other metabolic enzymes or transporters to simplify the structure of the PBPK model. CYP1A2 or CYP2C9 was primarily considered for inclusion in the modeling, the *K_m_* of CYP1A2 for 3′-methoxydadizein and 8-*O*-methylretusin were 44.408 and 5.409 μM, respectively; the *K_m_* of CYP2C9 for isolariciresinol was 16.423 μM, and the relevant *V_max_* was 9.308, 8.998, and 7.152 nM/min/nM, respectively. For daidzin, there were no predicted metabolic enzyme parameters, so the default values provided by the GastroPlus software were used eventually.

In the PBPK modeling, the tissue type was considered to be perfusion rate-limited, with the liver and kidneys identified as the primary organs for drug metabolism. Tissue-plasma partition coefficient (*K_p_*) is the ratio of drug concentration in tissue versus drug concentration in plasma and is an important parameter for drug distribution. The *K_p_* of tissues in rats was calculated using the Poulin and Theil equation that is in the built-in software of GastroPlus, with the assumption that these constituents enter tissues and plasma uniformly via passive diffusion, without specific binding to lipids or plasma proteins.

#### 4.2.2. The Construction of the PBPK Model

**Figure 10:**
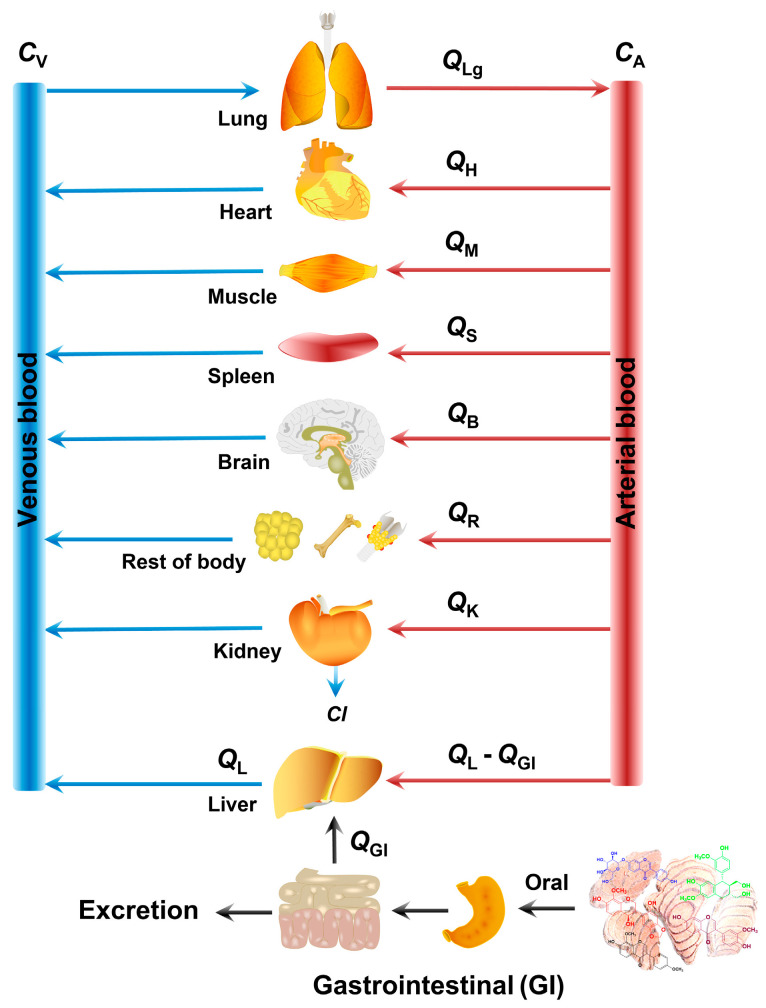
The physiological pharmacokinetic processes of the four constituents of SPC in rats by oral administration.

For the construction of PBPK models of these four active constituents of SPC in rats, assuming the drug is excreted mainly by the kidney and undergoes enterohepatic circulation, with blood flow rates in arteries and veins being the same and represented by Q, and the flow rates in each compartment as shown in Figure 10, the physiological pharmacokinetic process of these constituents in any given tissue can be calculated using a system of differential equations (Equation (1)). The PBPK models were constructed by GastroPlus software. In brief, the collected parameters of these constituents motioned in the previous section were all imported into GastroPlus software to simulate the concentration-time curves and gain the predicted pharmacokinetic parameters. The consistency between the calculated results (Calc.) and the observable values (Obs.) was then evaluated.

The system of differential equations was shown as follows:

(1) $$\left\{\begin{array}{c} C_{OB}=C_{i}/k_{i}\\ Lung:\;dC_{Lg}/dt={Q_{Lg}(C}_{V}-C_{Lg}/k_{Lg})/V_{Lg}\\ Liver:\;dC_{L}/dt={[(Q}_{L}-Q_{GI})C_{A}+Q_{GI}C_{GI}/k_{GI}-Q_{L}C_{L}/k_{L}-f_{b}V_{max}C_{L}/{(K}_{M}k_{L}+f_{b}C_{L})]/V_{L}\\ Kidney:\;dC_{K}/dt={(Q_{K}C}_{A}-{Q_{K}C_{K}/k_{K}-{Cl}^{\prime }}_{K}{C^{\prime }}_{K})/V_{K}\\ Heart:\;dC_{H}/dt=Q_{H}{(C}_{A}-C_{H}/k_{H})/V_{H}\\ Muscle:\;dC_{M}/dt=Q_{M}{(C}_{A}-C_{M}/k_{M})/V_{M}\\ Spleen:\;dC_{S}/dt=Q_{S}{(C}_{A}-C_{S}/k_{S})/V_{S}\\ Brain:\;dC_{B}/dt=Q_{B}{(C}_{A}-C_{B}/k_{B})/V_{B}\\ Rest\;of\;body:\;dC_{R}/dt=Q_{R}{(C}_{A}-C_{R}/k_{R})/V_{R}\\ Artery:\;dC_{A}/dt={Q(C_{Lg}/k_{Lg}-C}_{A})/V_{A}\\ Venous:\;dC_{V}/dt={(Q_{H}C}_{H}/k_{H}+Q_{M}C_{M}/k_{M}+{Q_{S}C_{S}/k_{S}+Q}_{B}C_{B}/k_{B}\\ {+{Q_{R}C_{R}/k_{R}+Q}_{K}C_{K}/k_{K}+Q}_{L}C_{L}/k_{L}-{Q_{Lg}C}_{V})/V_{V} \end{array}\right.$$

*C*_OB_ means the drug concentration in the blood when blood flows out from tissue. *C*_i_ means the drug concentration in tissue. *C*_A_ and *C*_v_ means the drug concentration in the artery and vein, respectively. *Cl*’ means clearance. *Q* means the blood flow rate of drug in tissues. *k*_i_ means the concentration ratio of tissue and blood. *V*_i_ means distribution volume of drug in tissue. *K*_M_ means Michaelis constant.

#### 4.2.3. Evaluation of the PBPK Model

The values of the PBPK model were linear regression to the observed values, and the correlation coefficient *R*^2^ was calculated simultaneously. The *R*^2^ of a qualified model with acceptable simulation effects should be no less than 0.8 [26,27]. Parameters including *C_max_*, *T_max_*, and *AUC* of each compound in the PBPK model were calculated and compared with the corresponding observable values. Fold-error (FE) of Cal. and Obs. or Val. were calculated using Equation (2), the model is accurately constructed when FE is less than 2 [28].

(2) $$\mathrm{FE}={10\;}^{|\log(\frac{\mathrm{Simulated}}{\mathrm{Observed}})|}$$

#### 4.2.4. Sensitivity Analysis

For the sensitivity analysis of parameters, we primarily focused on the solubility of the constituents and the parameters that influence their absorption in the human body. We selected seven parameters, mainly the particle size of the constituents, the permeability (*P_eff_*), solubility, *LogD*, *CL*, *R_bp_*, and *f_up_* of humans for sensitivity analysis, as the variations of these parameters not only affect the solubility and absorption of the constituents of SPC in vivo but also influence the distribution and metabolism. These factors, in turn, impact the accuracy of the PBPK model.

#### 4.2.5. Prediction of the Pharmacokinetics Among Different Doses and Species

After being constructed and verified, the PBPK models were used to predict the pharmacokinetic characteristics of these four constituents in rats at different oral doses and multiple doses administration, respectively.

The pharmacokinetic fitting results of rats given 60 g crude drug/kg body weight of SPC by gavage were verified by experiments. The verification experiment was conducted according to our previous pharmacokinetic study [16]. In brief, 1.5 mL suspension of SPC was given to rats by oral administration; 300 μL blood samples from orbital venous at 0.083, 0.25, 0.5, 0.75, 1.0, 1.5, 2, 3, 4, 6, 8, and 12 h were collected and pretreated with heparin sodium. After centrifugation, the plasma samples were obtained and further processed for subsequent detection by UFLC-MS/MS; the detailed analysis method is shown in Supplementary Data (Table S1 and Figure S1). The concentration-time curve of each compound was gained, and the pharmacokinetic parameters were calculated using DAS 2.0 software (DAS 2.0, Mathematical Pharmacology Professional Committee of China, Shanghai, China). The results of these four constituents were compared with the PBPK parameters predicted in rats at an oral dose of 60 g crude drug/kg body weight of SPC.

For the pharmacokinetic characteristics extrapolated from rats to humans, the *CL* in humans was predicted by a single species allometric scaling of rats (SSSrat) based on Equation (3) [39]; the distribution volume (*V_ss_*) was calculated according to Equation (4) using GastroPlus software. Therefore, the PBPK models of these four constituents in humans were constructed to predict the corresponding pharmacokinetic characteristics.

(3) $${\mathrm{CL}}_{u,\mathrm{human}}={\mathrm{CL}}_{u,\mathrm{rat}}\times {\left(\frac{W_{\mathrm{human}}}{W_{\mathrm{rat}}}\right)}^{0.75}$$

*CL_u_*_,*human*_ and *CL_u,rat_* represent clearance of humans and rats, respectively. *W_human_* and *W_rat_* represent the weight of humans and rats, respectively. In this model, an adult male weight of 70 kg and a rat weight of 0.25 kg were set as parameters for model prediction.

*V_ss_* = *V_p_* + *V_e_* × *E*/*P* + ∑*V_t_* × *K_p_* (4)

*V_p_* represents the volume of plasma; *V_e_* represents the volume of erythrocyte; *E/P* represents the ratio of erythrocyte to plasma concentration; *V_t_* was the volume of tissues; *K_p_* represents the partition coefficients of plasma.

## 5. Conclusions

In conclusion, our study established PBPK models of four active constituents from SPC successfully, and these models can accurately predict the pharmacokinetic behavior of whole herbs in different doses and different species. TCM is a system with complex chemical constituents; the application of PBPK models to predict the pharmacokinetic characteristics of different constituents of TCM may shed light on the problems of unclear dosages caused by multiple constituents in TCM in clinical practice.

## Figures and Tables

**Table 1:** The physicochemical and biochemical parameters of active constituents **1**–**4**.

Parameters	1	2	3	4	Source
*MW*	284.27	298.30	416.38	360.41	ChemDraw 21.0
*Log P*	2.676	2.805	0.541	1.591	ADMET Predictor
*pKa*	8.510	8.830	9.650	9.370	ADMET Predictor
*Log D*	2.640	2.850	0.560	2.020	GastroPlus
Solubility (mg/mL)	0.0205 (pH 6.32)	0.0108 (pH 6.60)	0.720 (pH 6.40)	0.201 (pH 6.17)	GastroPlus
*P_app_* (×10^−5^ cm/s)	0.220	2.480	0.400	0.220	Our study [24,25]
*R_bp_,_rat_*	0.915	0.875	1.044	0.830	ADMET Predictor
*R_bp_,_human_*	0.807	0.777	0.744	0.787	ADMET Predictor
*f_up_,_rat_*	8.403	11.816	39.933	31.535	ADMET Predictor
*f_up_,_human_*	7.394	7.07	17.891	16.139	ADMET Predictor
*CL_rat_* (L/h)	2.786	1.906	4.669	7.152	GastroPlus
*Vss_rat_* (L)	54.730	5.860	17.36	17.03	GastroPlus

**Table 2:** The *K_p_* values of constituents **1**–**4** from SPC in different rat tissues.

*K_p_*	1	2	3	4
Lung	0.45	2.42	0.48	1.25
Adipose	0.07	6.84	0.15	2.23
Muscle	0.39	1.19	0.36	0.70
Liver	0.37	2.05	0.37	1.04
Spleen	0.39	1.14	0.39	0.69
Heart	0.40	1.67	0.43	0.92
Brain	1.60	4.73	0.44	2.11
Kidney	0.73	1.92	0.42	1.02
Skin	1.09	2.72	0.47	1.35
ReproOrg	0.41	1.93	0.43	1.02
RedMarrow	0.33	2.51	0.31	1.17
YellowMarrow	0.07	6.84	0.15	2.23
Rest of body	0.42	1.16	0.40	0.71

**Table 3:** Comparisons of Obs. and Calc. values of four constituents from SPC in rats.

Constituents	Parameters	*C_max_* (μg/mL)	*T_max_* (h)	*AUC_0__→__t_* (μg∙h/mL)	*AUC_0__→__∞_* (μg∙h/mL)
**1**	Obs.	0.0039	0.42	0.0084	0.0090
	Calc.	0.0034	0.64	0.0092	0.0097
	FE	1.15	1.52	1.10	1.08
**2**	Obs.	0.0136	0.25	0.0097	0.0115
	Calc.	0.0130	0.20	0.0144	0.0146
	FE	1.05	1.25	1.48	1.27
**3**	Obs.	0.0151	0.21	0.0284	0.0305
	Calc.	0.0167	0.32	0.0196	0.0200
	FE	1.11	1.52	1.45	1.52
**4**	Obs.	0.0262	0.31	0.0347	0.0383
	Calc.	0.0247	0.32	0.0395	0.0403
	FE	1.06	1.03	1.14	1.05

**Table 4:** Comparisons of Calc. and Val. values of four constituents in rats at twice the oral dose of SPC.

Constituents	Parameters	*C_max_* (μg/mL)	*T_max_* (h)	*AUC_0__→__t_* (μg∙h/mL)	*AUC_0__→__∞_* (μg∙h/mL)
**1**	Calc.	0.0068	0.64	0.0183	0.0193
	Val.	0.0072	0.55	0.0191	0.0200
	FE	1.06	1.16	1.04	1.04
**2**	Calc.	0.0257	0.20	0.0288	0.0292
	Val.	0.0253	0.25	0.0153	0.0155
	FE	1.02	1.25	1.88	1.88
**3**	Calc.	0.0334	0.32	0.0393	0.0400
	Val.	0.0272	0.25	0.0355	0.0359
	FE	1.23	1.28	1.11	1.11
**4**	Calc.	0.0494	0.32	0.0791	0.0805
	Val.	0.0417	0.30	0.0557	0.0593
	FE	1.16	1.20	1.42	1.36

**Table 5:** The predicted parameters in humans of four constituents from SPC.

Constituents	*C_max_* (μg/mL)	*T_max_* (h)	*AUC_0__→__t_* (μg∙h/mL)	*AUC_0__→__∞_* (μg∙h/mL)
**1**	0.1578	5.76	1.635	6.650
**2**	0.0852	0.800	0.286	2.145
**3**	1.849	1.68	11.639	13.130
**4**	0.9699	1.97	6.359	13.601

## Data Availability

All data are presented in the article and Supplementary Materials.

## Supplementary Materials

The following supporting information can be downloaded at: https://www.mdpi.com/article/10.3390/ph17121621/s1, The instrument and analytical conditions of the UFLC-MS/MS method (Figure S1: MRM chromatograms of rat plasma; Table S1: The retention time and ion equivalent parameters of 4 constituents in rat plasma samples); The experimental C-t data of the five active compounds in rats for validation (Table S2: The experimental C-t data of the four active compounds in rats for validation); The experimental data in rats calculated by DAS 2.0.(Table S3: Compartment model parameters of 3′-methoxydaidzein; Table S4: Statistical moment parameters of 3′-methoxydaidzein; Table S5: Compartment model parameters of 8-*O*-methylretusin; Table S6: Statistical moment parameters of 8-*O*-methylretusin; Table S7: Compartment model parameters of Daidzin; Table S8: Statistical moment parameters of Daidzin; Table S9: Compartment model parameters of Isolariciresinol; Table S10: Statistical moment parameters of Isolariciresinol).
