# Supplementary material for: Predicting Pharmacokinetics of Active Constituents in Spatholobi caulis by Using Physiologically Based Pharmacokinetic Models
Source: Pharmaceuticals (Basel). 2024 Dec 3;17(12):1621. doi: 10.3390/ph17121621 (PMC11677527; doi:10.3390/ph17121621)
Supplement: Supplementary file 1 [file pharmaceuticals-17-01621-s001.zip › pharmaceuticals-3255532-supplementary.pdf]

1. The instrument and analytical conditions of UFLC-MS/MS method

1. Materials

4 constituents (**1**. 3′-methoxydadizein; **2**. 8-*O*-methylretusin; **3**. Daidzin; and **4**. Isolariciresinol) were all isolated from the aqueous extract of SPC with the purity > 98 % examined by HPLC and LC-MS in our previous study [16].

The internal standard (IS) carbamazepine with a purity of more than 99 % was purchased from Beijing JK-chemical technology Co. Ltd., (Beijing China). Heparin sodium was gained from Tianjin biochemical pharmaceutical Co. Ltd, (Tianjin, China). Ammonium acetate of HPLC grade, methanol and acetonitrile of LC-MS grade were purchased from Sigma (LOT# BCBK6717V, sigma-aldrich, Co., MO, USA) and Fisher (Fisher Corp., Fair lawn, NJ, USA) respectively. The experimental ultrapure water was prepared by Millipore Milli-Q ultrapure water preparation apparatus (Millipore Sigma, Bedford, MA, USA).

2. Methods

The detailed information of Shimadzu UFLC-MS/MS 8050 liquid mass system equipped with Nexera X2UFLC liquid chromatography system and 8050 triple quadrupole mass spectrometers with an electrospray ionization source were listed in our previous study [16]. The supporting LabSolution workstation was used for data acquisition.

The separation without superposition of analytes in plasma sample was achieved on a Kinetex® C18 chromatographic column (100mm×2.1mm i.d.; 2.6 µm, 100 Å; Phenomenex, Inc., Torrance, CA, USA) assembled with a Phenomenex p/n AJ0-9000 C18 protection column. The mobile phase was consisted of 0.5 mM ammonium acetate in water (**A**) and ACN (**B**). The gradient elution program was selected ultimately as follows: 5 to 7.5 % B (0–2.5 min), 7.5 to 10 % B (2.5–3 min), 10 to 18 % B (3–9 min), 18 to 28 % B (9–9.5 min), 28 to 55 % B (9.5–18.5 min), 55 to 95 % B (18.5–18.7 min), 95 % B (18.7–22 min). Column and automatic sampler temperature were set as 30 °C and 4 °C respectively. The flow rate of mobile phase was 0.35 mL/min while sample injection volume was 4 µL.

Multiple reaction monitoring (MRM) of positive or negative modes were used as scanning modes for the analytes and IS. Other essential parameters were successively optimized as: Nebulizing gas flow, 3 L/min; Heating gas flow, 10 L/min; Drying gas flow, 10 L/min; Interface temperature, 300 ºC; DL temperature, 250 ºC; Interface and detector voltage were each set as 3.0 kV and 1.8 kV. The detection parameters such as retention time (RT), dwell time (DT), quadrupole 1 pre-rod bias (Q1), collision energy (CE) and quadrupole 3 pre-rod bias (Q3) of these four compounds were shown in Table S1.

Table S1 The retention time and ion equivalent parameters of 4 constituents in rat plasma samples

Constituents	Retention time (min)	Precursor ion species	Precursor ion	Product ion	Dwell time (msec)	Q1 Pre Bias (V)	Collision energy (V)	Q3 Pre Bias (V)

**1**	11.653	[M−H] ⁻	283.35	268.10	11	20	18	28
**2**	12.731	[M−H] ⁻	297.20	282.10	11	16	17	30
**3**	7.260	[M+H] ⁺	417.15	255.10	33	-23	-22	-28
**4**	9.714	[M−H] ⁻	359.20	344.30	69	19	19	23

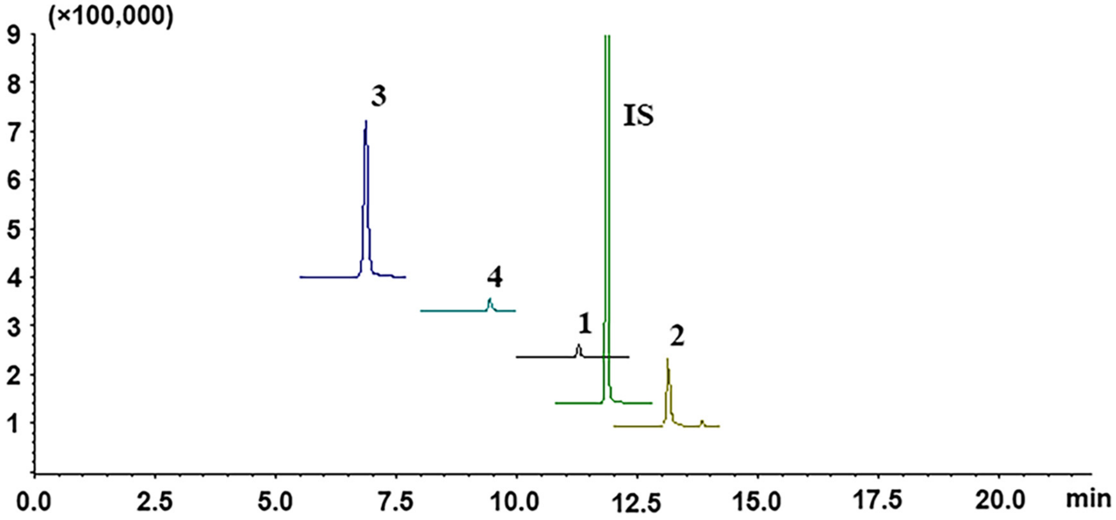

Figure S1 MRM chromatograms of rat plasma containing 4 constituents (**1**. 3′-methoxydadizein; **2**. 8-*O*-methylretusin; **3**. Daidzin; and **4**. isolariciresinol) of SPC and IS (Carbamazepine) collected at 1 h after oral administration

2. The experimental C-t data of the five active compounds in rats for validation

Compounds: 3′-methoxydaidzein (**1**), 8-*O*-methylretusin (**2**), daidzin (**3**) and isolariciresinol (**4**)

Unit of concentration: ug/L

Unit of time: h

Lowest limits of detection: 0.001

Parallel rats: 5

Table S2. The experimental C-t data of the four active compounds in rats for validation

Compounds	**1**		**2**		**3**		**4**	
Dose	0.124 mg/kg		0.22 mg/kg		1.4 mg/kg		2.62 mg/kg	
Time	Mean	SD	Mean	SD	Mean	SD	Mean	SD
0.083	2.984	0.366	8.998	1.609	16.353	2.950	15.411	3.577
0.25	5.517	1.017	22.891	3.288	27.965	4.115	41.486	4.540
0.5	7.088	0.873	12.327	2.200	20.989	1.379	41.744	1.146
0.75	6.157	0.412	5.226	0.855	18.435	1.397	27.630	1.496
1	5.774	0.350	3.331	0.987	10.881	1.321	22.189	2.963
1.5	5.107	0.264	1.966	0.397	5.551	0.708	8.287	1.380
2	2.894	0.559	0.809	0.103	3.156	0.740	7.032	0.884
3	1.749	0.066	0.809	0.103	2.619	0.745	4.267	1.143
4	1.455	0.184	0.409	0.166	1.565	0.188	3.409	0.613
6	0.553	0.114	0.445	0.235	1.394	0.243	2.012	0.916
8	0.582	0.042			0.161	0.042	1.175	0.263
12	0.197	0.114			0.271	0.109		

3. The experimental data in rats calculated by DAS 2.0

3′-methoxydaidzein (**1**)

Dose: 0.124 mg/kg

Unit of concentration: ug/L

Unit of time: h

Lowest limits of detection: 0.001

Parallel rats: 5

Table S3. Compartment model parameters of 3′-methoxydaidzein (**1**)

parameters	units	Mean	SD
t1/2α	h	1.009	0.17
t1/2β	h	14.423	15.527
V1/F	L/kg	12.821	0.71
CL/F	L/h/kg	6.06	0.162
AUC(0-t)	ug/L*h	19.088	0.616
AUC(0-∞)	ug/L*h	20.474	0.534
K10	1/h	0.474	0.036
K12	1/h	0.207	0.113
K21	1/h	0.135	0.064
Ka	1/h	4.239	1.338
t1/2Ka	h	0.185	0.088
Tlag	h	0	0

Table S4. Statistical moment parameters of 3′-methoxydaidzein (**1**)

parameters	units	Mean	SD
AUC(0-t)		19.127	0.655
AUC(0-∞)	ug/L*h	20.023	0.608
AUMC(0-t)	ug/L*h	52.953	2.892
AUMC(0-∞)		68.893	12.561
MRT(0-t)		2.771	0.177
MRT(0-∞)	h	3.438	0.605
VRT(0-t)	h	7.148	1.03
VRT(0-∞)	h^2	16.239	7.081
t1/2z	h^2	2.9	0.577
Tmax	h	0.55	0.112
CLz/F	h	6.197	0.19
Vz/F	L/h/kg	25.899	4.944
Zeta	L/kg	0.247	0.048
$Zeta_{TP}$		--	--
Cz		0.202	0.076
Cmax	ug/L	7.179	0.629

8-*O*-methylretusin (**2**)

Dose: 0.22 mg/kg

Unit of concentration: ug/L

Unit of time: h

Lowest limits of detection: 0.001

Parallel rats: 5

Table S5. Compartment model parameters of 8-*O*-methylretusin (**2**)

parameters	units	Mean	SD
t1/2α	h	34.861	39.784
t1/2β	h	35.422	39.142
V1/F	L/kg	12.043	17.445
CL/F	L/h/kg	12.552	5.493
AUC(0-t)	ug/L*h	19.779	11.091
AUC(0-∞)	ug/L*h	22.361	15.206
K10	1/h	6.671	10.483
K12	1/h	0.394	0.665
K21	1/h	0.474	0.495
Ka	1/h	2.056	2.947
t1/2Ka	h	20.246	33.118
Tlag	h	0	0

Table S6. Statistical moment parameters of 8-*O*-methylretusin (**2**)

parameters	units	Mean	SD
AUC(0-t)	ug/L*h	15.278	0.75
AUC(0-∞)	ug/L*h	15.459	0.626
AUMC(0-t)		15.37	1.806
AUMC(0-∞)		20.163	3.617
MRT(0-t)	h	1.006	0.1
MRT(0-∞)	h	1.301	0.195
VRT(0-t)	h^2	1.65	0.29
VRT(0-∞)	h^2	3.316	0.804
t1/2z	h	1.067	0.264
Tmax	h	0.25	0
CLz/F	L/h/kg	14.25	0.587
Vz/F	L/kg	22.09	6.426
Zeta		0.675	0.136
$Zeta_{TP}$		--	--
Cz	ug/L	0.107	0.055
Cmax	ug/L	25.268	5.46

Daidzin (**3**)

Dose: 1.4 mg/kg

Unit of concentration: ug/L

Unit of time: h

Lowest limits of detection: 0.001

Parallel rats: 5

Table S7. Compartment model parameters of Daidzin (**3**)

parameters	units	Mean	SD
t1/2α	h	0.868	0.837
t1/2β	h	36.545	37.84
V1/F	L/kg	37.289	8.031
CL/F	L/h/kg	35.643	2.966
AUC(0-t)	ug/L*h	37.149	3.274
AUC(0-∞)	ug/L*h	39.487	3.339
K10	1/h	0.981	0.168
K12	1/h	0.402	0.307
K21	1/h	0.15	0.122
Ka	1/h	16.131	20.518
t1/2Ka	h	0.385	0.613
Tlag	h	0	0

Table S8. Statistical moment parameters of Daidzin (**3**)

parameters	units	Mean	SD
AUC(0-t)	ug/L*h	35.516	2.412
AUC(0-∞)	ug/L*h	35.92	2.394
AUMC(0-t)		65.207	7.221
AUMC(0-∞)		75.236	7.711
MRT(0-t)	h	1.833	0.11
MRT(0-∞)	h	2.092	0.113
VRT(0-t)	h^2	4.934	0.584
VRT(0-∞)	h^2	8.138	1.86
t1/2z	h	1.822	0.727
Tmax	h	0.25	0
CLz/F	L/h/kg	39.111	2.689
Vz/F	L/kg	103.062	41.542
Zeta		0.429	0.165
$Zeta_{TP}$		--	--
Cz	ug/L	0.117	0.122
Cmax	ug/L	27.183	4.225

Isolariciresinol (**4**)

Dose: 2.62 mg/kg

Unit of concentration: ug/L

Unit of time: h

Lowest limits of detection: 0.001

Parallel rats: 5

Table S9. Compartment model parameters of Isolariciresinol (**5**)

parameters	units	Mean	SD
t1/2α	h	0.433	0.121
t1/2β	h	17.429	29.061
V1/F	L/kg	29.573	11.601
CL/F	L/h/kg	99.561	130.836
AUC(0-t)	ug/L*h	46.421	24.823
AUC(0-∞)	ug/L*h	52.808	25.446
K10	1/h	7.512	14.088
K12	1/h	0.448	0.297
K21	1/h	0.247	0.111
Ka	1/h	5.032	1.943
t1/2Ka	h	0.175	0.123
Tlag	h	0	0

Table S10. Statistical moment parameters of Isolariciresinol (**5**)

parameters	units	Mean	SD
AUC(0-t)	ug/L*h	55.749	4.388
AUC(0-∞)	ug/L*h	59.323	3.407
AUMC(0-t)		82.484	10.038
AUMC(0-∞)		123.911	24.641
MRT(0-t)	h	1.479	0.126
MRT(0-∞)	h	2.084	0.363
VRT(0-t)	h^2	2.067	0.272
VRT(0-∞)	h^2	6.027	1.669
t1/2z	h	1.727	0.293
Tmax	h	0.3	0.112
CLz/F	L/h/kg	44.282	2.557
Vz/F	L/kg	111.04	23.613
Zeta		0.412	0.078
$Zeta_{TP}$		--	--
Cz	ug/L	1.39	0.352
Cmax	ug/L	41.698	4.591
